# Identification of Submergence Tolerance Loci in Dongxiang Wild Rice (DXWR) by Genetic Linkage and Transcriptome Analyses

**DOI:** 10.3390/ijms26051829

**Published:** 2025-02-20

**Authors:** Jilin Wang, Cheng Huang, Lijuan Tang, Hongping Chen, Ping Chen, Dazhou Chen, Dianwen Wang

**Affiliations:** Rice National Engineering Research Center (Nanchang), Rice Research Institute, Jiangxi Academy of Agricultural Sciences, Nanchang 330200, China; wangjilin1982@163.com (J.W.); chenghuang@webmail.hzau.edu.cn (C.H.); t1257601267@163.com (L.T.); 13970920363@139.com (H.C.); cp87090379@163.com (P.C.); cdz288@163.com (D.C.)

**Keywords:** Dongxiang wild rice, submergence tolerance, quantitative trait locus, transcriptomics, differentially expressed genes

## Abstract

The submergence tolerance of rice is a key factor in promoting rice direct seeding technology and resisting flood disasters. Dongxiang wild rice (DXWR) has strong submergence tolerance, but its genetic basis is still unclear. Here, we report quantitative trait loci (QTLs) analysis for hypoxic germination rate (HGR), hypoxic seedling rate (HSR), budlet submergence survival rate (BSSR) and seedling submergence survival rate (SSSR) using a linkage map in the backcross recombinant inbred lines (BRILs) that were derived from a cross of DXWR, and an indica cultivar, GZX49. A total of 20 QTLs related to submergence tolerance of rice were detected, explaining phenotypic variations ranging from 2% to 8.5%. Furthermore, transcriptome sequencing was performed on the seeds and seedlings of DXWR before and after submergence. During the seed hypoxic germination and seedling submergence stages, 6306 and 3226 differentially expressed genes (DEGs) were detected respectively. Gene ontology (GO) and Kyoto encyclopedia of genes and genomes (KEGG) analyses were conducted on these differentially expressed genes. Using genetic linkage analysis and transcriptome data, combined with qRT-PCR, sequence comparison, and bioinformatics, *LOC_Os05g32820* was putatively identified as a candidate gene for *qHGR5.2* co-located with HGR and SSSR. These results will provide insights into the mechanism of rice submergence tolerance and provide a basis for improving rice submergence tolerance.

## 1. Introduction

Rice (*Oryza sativa* L.), as one of the most important food crops in the world, plays an indispensable role in ensuring food security [1]. In recent years, frequent outbreaks of extreme weather have caused varying degrees of damage to rice production. Meanwhile, global warming has led to frequent floods. Therefore, flooding stress has become one of the major abiotic stresses in rice production [2,3]. Simultaneously, a substantial decline in rural labor force has occurred, resulting in a shortage of human resources. Rice direct seeding, which has adjusted to the present circumstances, is being extensively popularized. In addition, due to the significant increase in the number of grains per spike, the number of spikes per unit area, and the thousand grain weight, it shows a significant yield advantage over transplanted rice [4]. However, the levelness of mechanized farmland fails to meet the standards of paddy fields, and the importance of flood tolerance during seed germination, hypoxic germination ability, has become increasingly prominent. Improving the submergence tolerance of rice has become an important goal in rice breeding [5].

Rice submergence tolerance is controlled by multiple quantitative trait locu (QTLs) and is greatly influenced by the environment. Due to different treatment methods and evaluation indicators, a large number of submergence tolerance-related QTLs have been reported [6,7,8]. *CIPK15* is an important gene for anaerobic germination in rice. Under anaerobic conditions, the cipk15 mutant of rice cannot germinate or activate the transcription of α-amylase, while exogenous sucrose addition can restore the *cipk15* mutant to the normal phenotype, indicating that under anaerobic conditions, *CIPK15* is necessary for the expression of α-amylase and subsequent starch hydrolysis into sugar [9]. In addition, under conditions of insufficient sugar and oxygen, CIPK15 can regulate the accumulation of *SnRK1A* (Snf1 protein kinase) protein [9]. SnRK1A activates *MYBS1* protein through phosphorylation [10], thereby promoting the binding of MYBS1 to downstream specific elements to reactivate α-amylase [11]. It can also promote the interaction between MYBS1 and GAMYB (Gibberellin myb) to form a complex that enters the nucleus and coordinates the expression of downstream genes, including α-amylase genes [12]. In addition, Trehalose 6-phosphate (T6P) acts as a negative feedback regulator of sucrose levels in plants; the T6P/sucrose ratio is a key steady-state parameter in sugar signaling [13]. During seed germination, the Trehalose-6-Phosphate Phosphatase gene (*OsTPP7*) catalyzes the conversion of 6-phosphate trehalose (T6P) to trehalose, relieving T6P’s inhibition on SnRK1A [13,14]. At the same time, some factors downregulate the T6P/sucrose ratio, allocating energy from the endosperm reserve to the embryo sheath and promoting enhanced growth of the embryo sheath during anaerobic periods [14]. Under flooded conditions, the growth ability of rice embryo sheaths is crucial for their seedling formation. Researchers used association analysis to clone a flood tolerant major gene, *OsUGT75A*, which promotes rapid growth of embryo sheaths under flooded conditions by regulating the dynamic balance of abscisic acid (ABA) and jasmonic acid (JA) [15]. In addition, researchers have also detected some QTLs related to anaerobic germination in genetic populations constructed using rice varieties with strong and weak anaerobic germination abilities [16,17,18,19,20,21,22,23,24].

In the case of flooding, most rice varieties elongate their leaves to obtain oxygen from the water surface. However, some indica rice varieties can survive by inhibiting growth until the floodwaters recede, a strategy known as the “static strategy” [25]. The flood—tolerant rice variety containing *Sub1A*, a rice submergence tolerant gene, synthesizes ethylene under flooding conditions, promotes ABA degradation, and the expression of *Sub1A*. *Sub1A* promotes the accumulation of inhibitory factors *SLR1* and *SLRL1* in GA signaling, thereby inhibiting GA response. The GA response inhibits the elongation of plant’s aboveground parts underwater, reduces carbohydrate consumption, and ultimately improves flood resistance [26]. Overexpression of *SUB1A* enhances the response to ABA, thereby activating the expression of stress-induced genes [27]. Through map-based cloning, two submergence tolerance genes, *SK1* and *SK2*, which encode ERF transcription factors in deepwater rice, were cloned [28]. The expression of *SK1/SK2* is induced by submergence and ethylene, and their overexpression promotes internode elongation in normal paddy rice. *EIL1* in rice positively regulates the ethylene response and can activate the expression of *SK1/SK2*. There are amino acid differences in *SD1* between the deepwater rice variety C9285 and normal paddy rice, resulting in different enzymatic activities of SD1 [29]. *EIL1* can activate *SD1* to promote GA accumulation, especially GA4. In deepwater rice, there is an antagonistic regulatory mechanism for GA-regulated stem growth. *ACE1* endows the cells in the intercalary meristematic region with the competence for cell division, leading to internode elongation, while *DEC1* suppresses it [30].

RNA-sequencing, which is proficient in delineating the genome-wide gene expression panorama, represents a highly effective avenue for discerning stimuli responsive genes throughout the entire genome [31]. Through transcriptome sequencing analysis, a large number of genes responsive to submergence stress have been identified in rice. These genes involve multiple regulatory pathways such as hydrolase activity, biosynthesis of secondary metabolites, signal transduction and transcriptional regulation [32,33,34,35,36]. Dongxiang wild rice (DXWR), known as the “giant panda of plants”, has extremely strong flood tolerance. Its flood tolerance at the seedling stage is even stronger than that of FR13A, which is currently recognized as the most submergence-tolerant variety. However, the genetic mechanism of submergence tolerance in DXWR remains unclear.

In this study, the backcross recombinant inbred lines (BRILs) population [37] constructed by using DXWR and GZX49 was employed to conduct QTL analysis on submergence tolerance-related traits. Transcriptome sequencing was carried out on the hypoxic germination of DXWR and the flooding of seedlings, and the differentially expressed genes were analyzed. Candidate gene analysis was performed on the QTL (*qHGR5.2*) that was co-located for the hypoxic germination rate and the survival rate of seedlings under submergence. These findings provide new insights into the genetic basis of submergence tolerance in rice.

## 2. Results

### 2.1. Analysis of Submergence Tolerance Phenotype

**Figure 1:**
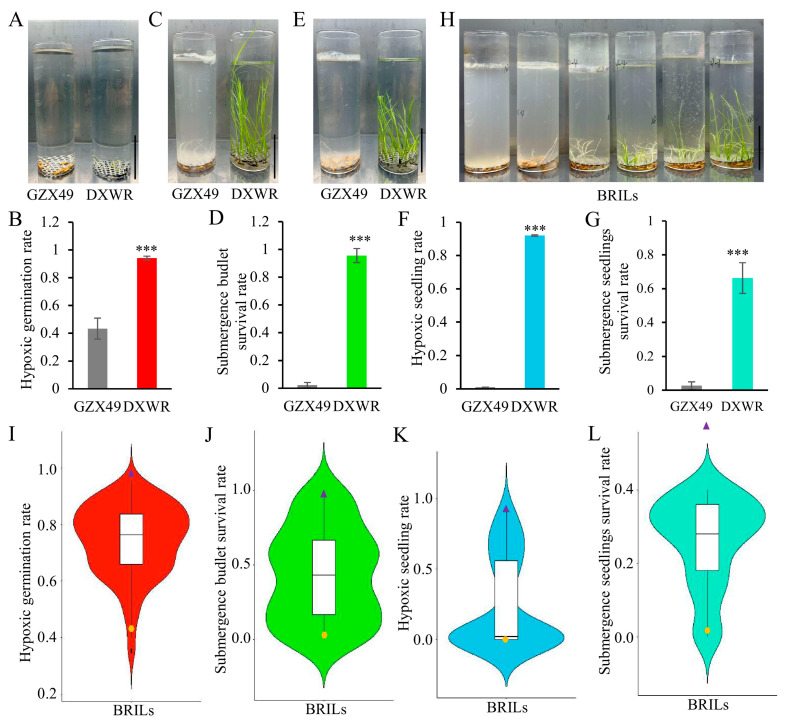
Analysis of submergence tolerance phenotypes. (**A**–**G**) show the submergence tolerance phenotypes of Dongxiang wild rice (DXWR) and GZX49. (**A**,**C**,**E**) are the phenotypic diagrams of the hypoxic germination rate (HGR), the submergence budlet survival rate (BSSR), and the hypoxic seedling rate (HSR) for DXWR and GZX49, respectively. Differences between DXWR and GZX49 in HGR (**B**), BSSR (**D**), HSR (**F**) and submergence seedlings survival rate (SSSR) (**G**). Data are given as the mean and standard error (*n* = 3 individuals). The results were statistically analyzed using Student’s *t*-test (*** *p*  <  0.001). (**H**) Phenotypes of some lines of the BRIL population HGR. The scale bar of (**A**,**C**,**E**,**H**) means 5 cm. (**I**–**L**) Violin plot of phenotype variation related to submergence tolerance in BRILs. The box-plot part in the middle contains information such as the median, upper and lower quartiles. The upper and lower boundaries of the box are the upper quartile and the lower quartile, respectively, and the horizontal line in the middle of the box represents the median. The “violin” parts on both sides indicate the density of the data at that position. The wider a “violin” part is, the more concentrated the data distribution is in the corresponding area, while the narrower it is, the sparser the distribution is. The yellow dots and purple triangles represent the values of the phenotypes of the population parents GZX49 and DXWR, respectively.

Wild rice has stronger stress resistance compared to cultivated rice. A survey on the flood resistance of DXWR and GZX49 found that among the four submergence tolerance indicators at different stages, hypoxic germination rate (HGR), budlet submergence survival rate (BSSR), hypoxic seedling rate (HSR) and seedlings submergence survival rate (SSSR), DXWR was significantly stronger than GZX49. The hypoxic germination rate of DXWR reached 95%, while GZX49 only had 41% (Figure 1A,B). The budlet submergence survival rate and hypoxic seedling rate of GZX49 were almost 0, while DXWR still reaches over 90% (Figure 1C–F). Similarly, after flooding treatment of seedlings, the survival rate of GZX49 remained 0, while DXWR still had 65% (Figure 1G). These results indicate that DXWR has a strong hypoxic germination ability and seedling flooding tolerance compared to GZX49.

The backcross recombinant inbred lines (BRILs) population, which was developed with GZX49 as the recipient parent and DXWR as the donor and backcrossed with the same parent for one generation, exhibits extensive variation in four submergence tolerant phenotypes at different stages. The phenotype values of four traits related to submergence tolerance in the recombinant inbred line populations for these traits: HGR, BSSR, HSR, and SSSR are all between the two parents, and there is no strain with stronger submergence tolerance than DXWR (Figure 1H–L). This indicates that the genetic basis of submergence tolerance in DXWR is relatively complex, with multiple QTLs related to submergence tolerance, and there is genetic interaction between these QTLs.

Correlation analysis was conducted on the four submergence tolerance phenotype values of the population at different stages, and the results showed that there was a certain correlation between the four traits. HGR is significantly positively correlated with HSR, BSSR is significantly correlated with SSSR, and SSSR is significantly correlated with HSR (Figure S1). This indicates that the submergence tolerance traits at different stages have a common genetic basis, meaning that DXWR has loci that enhance submergence tolerance during germination and seedling growth.

### 2.2. QTL Analysis of Submergence Tolerance

**Figure 2:**
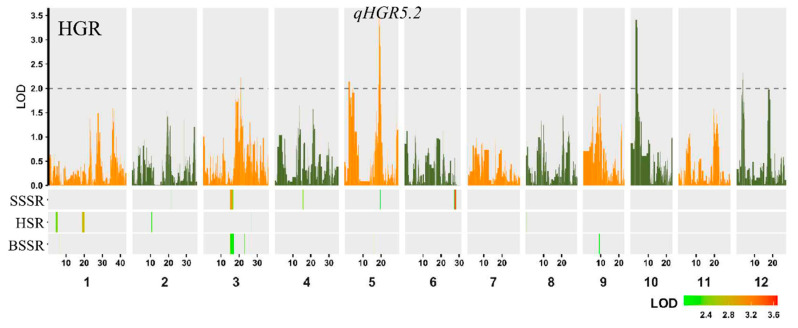
Overview of QTLs and significant SNPs related to four submergence tolerance phenotypes. The top graph (Manhattan plot) shows logarithm of odds (LOD) scores for hypoxic germination rate (HGR). Horizontal dashed lines in the plots indicate the declaration thresholds. The results of three traits seedling submergence survival rate (SSSR), hypoxic seedling rate (HSR) and budlet submergence survival rate (BSSR) are presented below. Rectangle color density indicates the magnitude of −log10 (*p*) values in the ridge regression significance test. The horizontal position of the rectangle indicates the relative physical position of QTL support interval on the chromosome.

The ridge regression analysis for QTL detection was performed in the BRIL population using the bin genotypes, which has been previously reported in detail [37]. A total of 20 QTLs were detected for 4 submergence tolerance traits at different stages (Figure 2 and Table 1). This study detected 5 QTLs that affect HGR, which are located on chromosomes 3, 5, 10, and 12, explaining 31.6% of the population phenotype variation. The largest effect is *qHGR5.2*, with an additive effect of 0.039, explaining 8.4% of the phenotypic variation. Five QTLs related to BSSR were detected, with positive allelic effects of DXWR, which are located on chromosomes 1, 3, 5, and 9, explaining 24.1% of the population’s phenotypic variation. None of the five QTLs related to BSSR explained a phenotypic variation rate exceeding 6%, indicating that BSSR is controlled by multiple minor QTLs and is greatly influenced by the environment. Five HSR-related QTLs were detected, which are located on chromosomes 1, 2, 3, and 8, explaining 20.4% of the population phenotype variation. Similarly, there were 5 QTLs that affect SSSR, which are located on chromosomes 2, 3, 4, 5, and 6, accounting for 32.6% of the population phenotype variation, and 2 QTLs have phenotypic variation explained (PVE) greater than 8%. There are also co-significant bin intervals between the flood tolerance QTLs at four different stages, such as *qHGR5.2* and *qSSSR5*, which are significant at bin0806–0807 and have the same positive additive effect, indicating that these two QTLs are highly likely to be from the same locus.

### 2.3. Transcriptome Analysis of DEGs in DXWR Under Submergence Tolerance

**Figure 3:**
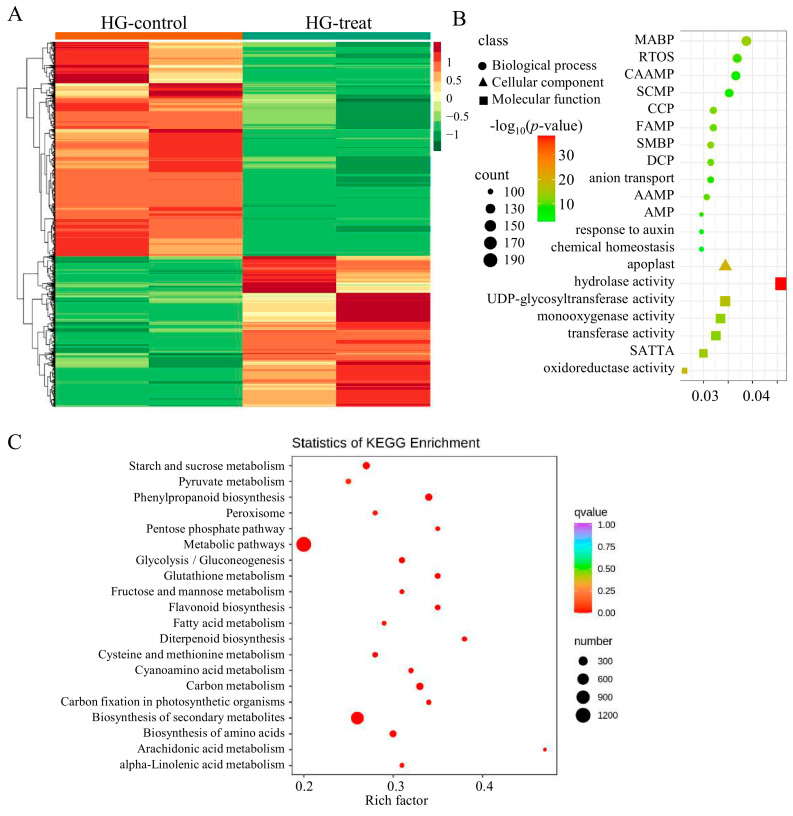
Transcriptome analysis of the genetic mechanism of DXWR in response to hypoxic germination. (**A**) The expression patterns of 6072 DEGs detected by transcriptome in DXWR during hypoxic germination response. The red color represents higher expression and the green color represents lower expression. Clustering was performed among both selected genes and across different samples, while sample identity is annotated at the top of each column. HG-control and HG-treat represent the blank control sample and treated sample of hypoxic germination, respectively. Parts (**B**,**C**) are gene ontology (GO) functional enrichment histogram and Kyoto encyclopedia of genes and genomes (KEGG) pathway enrichment histogram, respectively. MABP, RTOS, CAAMP, SCMP, CCP, FAMP, SMBP, DCP, AAMP, AMP and SATTA in (**B**) represent monocarboxylic acid biosynthetic process, response to oxidative stress, cellular amino acid metabolic process, sulfur compound metabolic process, carbohydrate catabolic process, fatty acid metabolic process, secondary metabolite biosynthetic process, drug catabolic process, alpha-amino acid metabolic process, antibiotic metabolic process and secondary active transmembrane transporter activity, respectively.

In order to identify the genes that respond to anaerobic germination in DXWR, we conducted hypoxic germination on fresh, plump, and dormant DXWR seeds. Samples taken after 6 and 24 h of hypoxic germination were used as blank controls and hypoxic treated samples, respectively, and transcriptome sequencing was performed. A total of 6072 DEGs were detected, of which 3644 were down-regulated, accounting for about 60%, and only 2428 were up-regulated (Figure 3A). Furthermore, the DEGs were classified according to gene ontology (GO) and Kyoto encyclopedia of genes and genomes (KEGG) terms. The most significant 20 GO clustering pathways involve 13 biological processes (BP), 6 molecular functions (MF), and 1 cellular component (CC), indicating that BP and MF pathways play important roles in the adaptation of DXWR to anaerobic environments for germination (Figure 3B). The *p*-value is the most significant because the hydrolase activity pathway contains 195 genes, followed by the UDP-glycosyltransferase activity pathway, which contains 146 genes (Figure 3B). It has been reported that UDP-glucosyltransferase encoding gene (*OsUGT75A*) mediates crosstalk between ABA and JA signaling functions in flooded environments, regulates coleoptile elongation, and enhances rice submerged tolerance [15]. The KEGG enrichment analysis of DEGs showed that 1220 DEGs were enriched in metabolic pathways and 812 genes were enriched in biosynthesis of secondary metabolites. These two pathways have the most enriched DEGs, and the *q* value is also the most significant (Figure 3C). These results indicate that DXWR can germinate smoothly in low oxygen environments by regulating the gene expression of metabolic and secondary metabolic pathways represented by hydrolase activity and UDP-glucosyltransferase activity.

**Figure 4:**
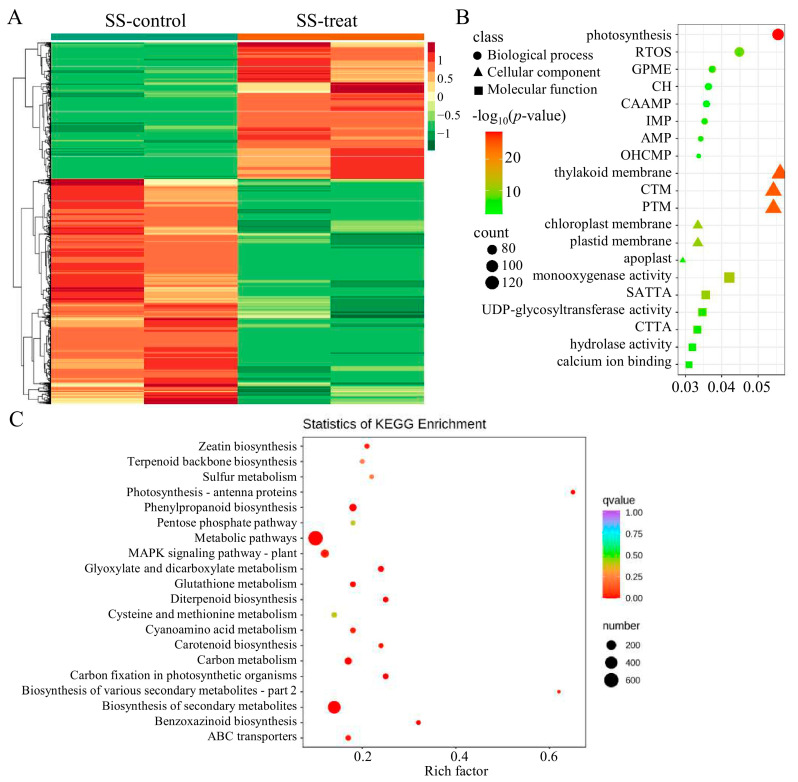
Transcriptome analysis of the genetic mechanism of DXWR seedling in response to submergence tolerance. (**A**) The expression patterns of 3074 DEGs detected by transcriptome in DXWR seedling in response to submergence tolerance. The red color represents higher expression and the green color represents lower expression. Clustering was performed among both selected genes and across different samples, while sample identity is annotated at the top of each column. SS-control and SS-treat represent the blank control sample and treated sample of submergence, respectively. Parts (**B**,**C**) are gene ontology (GO) functional enrichment histogram and Kyoto encyclopedia of genes and genomes (KEGG) pathway enrichment histogram, respectively. RTOS, GPME, CH, CAAMP, IMP, AMP, OHCMP, CTM, PTM, SATTA and CTTA in (**B**) represent response to oxidative stress, generation of precursor metabolites and energy, chemical homeostasis, cellular amino acid metabolic process, isoprenoid metabolic process, antibiotic metabolic process, organic hydroxy compound metabolic process, chloroplast thylakoid membrane, plastid thylakoid membrane, secondary active transmembrane transporter activity and cation transmembrane transporter activity, respectively.

Similarly, we conducted transcriptome sequencing analysis on DXWR seedlings before and after flooding, and detected a total of 3074 DEGs, including 1179 up-regulated genes and 1895 (61.6% of the total) down-regulated genes (Figure 4A). GO clustering was performed on DEGs, and the 20 pathways with the most significant *p*-values mainly 8 BPs, 6 MFs, and 6 CCs. Among the top four pathways with the most significant *p*-values, CC accounted for three, indicating that CC also plays an important role in seedling resistance to flooding (Figure 4B). The pathway with the most significant *p*-value is photosynthesis, which enriched 104 genes, indicating significant changes in gene expression on the photosynthesis pathway of DXWR under flooding conditions. This suggests that altering the photosynthesis mechanism is crucial for DXWR seedlings to resist flooding stress. KEGG enrichment analysis of DEGs showed that the DEGs responsive to flooding stress in seedlings were consistent with those in hypoxic germination, and the pathways with the highest number of enriched differential genes were still metabolic pathways (in which 615 DEGs were enriched) and biosynthesis of secondary metabolites (in which 440 DEGs were enriched) (Figure 4C), indicating that DXWR has the same mechanism for resisting submergence response at different stages.

### 2.4. Candidate Gene Analysis for qHGR5.2

**Figure 5:**
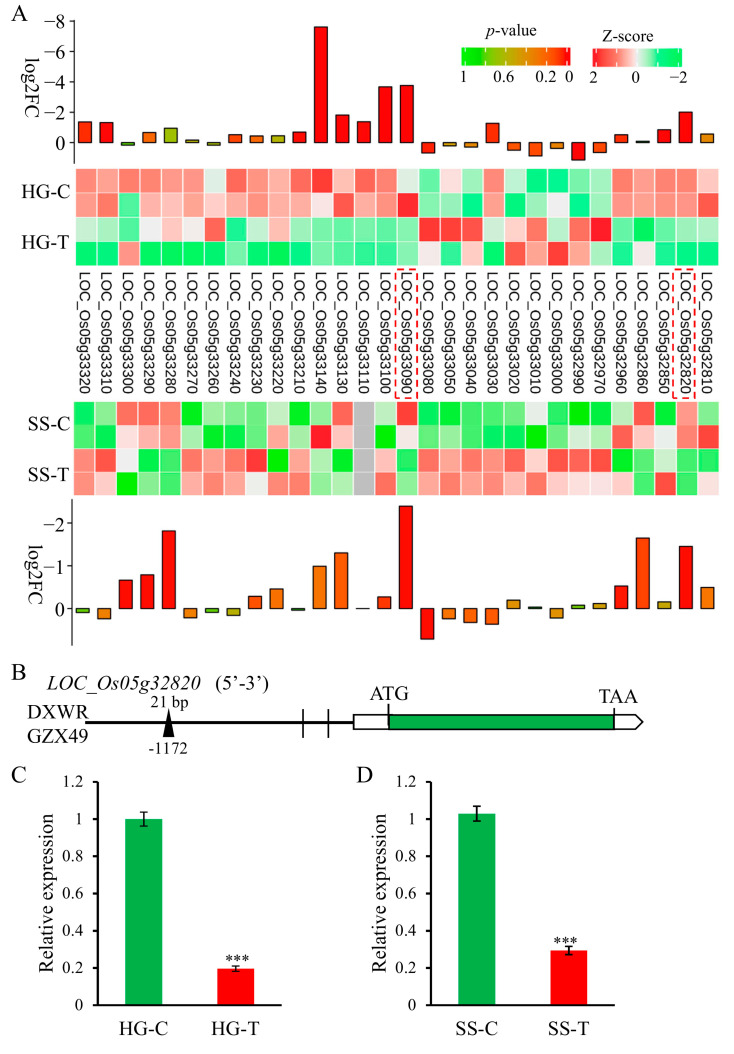
*qHGR5.2* candidate gene analysis. (**A**) Clustering heat maps of the relative expression levels of genes within the *qHGR5.2* localization interval determined using RNA-seq data. Standard-scores (Z-scores) were used as the numerical signs to evaluate the standard deviations from the mean of the corresponding samples. HG-C, HG-T, SS-C and SS-T represent the control sample of hypoxic germination, the treatment sample of hypoxic germination, the control sample of seedling flooding treatment and the treatment sample of seedling flooding, respectively. (**B**) Sequence comparison of *LOC_Os05g32820* between GZX49 and DXWR. Vertical bars and black triangles represent SNPs and Indel, respectively. Parts (**C**,**D**) represent the expression level of the *LOC_Os05g32820* in DXWR before and after hypoxic germination and seedling submergence by qRT-PCR, respectively. The results were statistically analyzed using Student’s *t*-test (*** *p*  <  0.001). Transcription levels relative to control, which was set at 1, are presented as the mean and of triplicates. *LOC_Os03g13170* (*Ubiquitin*) is the control gene.

In order to explore novel genes regulating hypoxic germination and seedling submergence tolerance, we selected a QTL co-located between HGR and SSSR, *qHGR5.2* (*qSSSR5*). The most significant bins for *qHGR5.2* in HGR and SSSR are bin0806 and bin0807, respectively. Therefore, we included bin0806 and bin0807 as the confidence intervals for *qHGR5.2*, which are 347 kb in size. Using the Rice Genome Annotation Project (uga.edu), it was found that interval of *qHGR5.2* contains about 56 genes. Using transcriptome sequencing data from hypoxic germination and seedling flooding of DXWR, we found 20 and 19 genes expressed in germination and seedling tissues, respectively, within the confidence interval of *qHGR5.2*. There are 10 DEGs during the low oxygen germination stage, including 9 down regulated expression genes and 1 up-regulated expression gene. However, only three genes responded to flooding stress during the seedling stage, and all of them were down-regulated in expression (Figure 5A). In the DEGs of low oxygen germination and seedling flooding treatment, *LOC_Os05g33090* and *LOC_Os05g32820* were significantly down-regulated in both (Figure 5A). *LOC_Os05g33090* encodes an expressed protein without GO annotation information, so *LOC_Os05g33090* is not considered as a candidate gene for *qHGR5.2*. *LOC_Os05g32820* is a peptide-N4-asparagine amidase A, also known as PNAase A, which is an enzyme involved in the post-translational modification process of proteins. This enzyme plays a specific role in the glycosylation modification pathway of proteins. It can catalyze the hydrolysis reaction of amide bonds on certain asparagine residues, which may affect the structure, function, stability, localization, and interactions of proteins within cells. Further analysis of the sequence variation of *LOC_Os05g32820* in GZX49 and DXWR revealed no variation in the coding region, but two SNPs and one indel were present in the promoter region. This indel contains 35 bp (there is an ACACTTGTATTTATGTTAGAATTTTACGTTTTTCC insertion in *LOC_Os05g32820*^GZX49^), located 1172 bp upstream of the initiator codon (Figure 5B). The expression of the gene LOC_Os05g32280 in DXWR before and after hypoxic germination and seedling submergence was further analyzed using qRT-PCR technology. The results showed that the expression of *LOC_Os05g32280^DXWR^* was significantly down-regulated both during hypoxic germination and seedling submergence, and this trend was consistent with that of the transcriptome analysis (Figure 5C,D). Element analysis was conducted on the 35 bp sequence on PlantPAN 4.0 (PlantPAN 4.0 (https://plantpan.itps.ncku.edu.tw/plantpan4/index.html (accessed on 20 November 2024))) [38], and the results showed that 35 bp contains a *bZIP* binding element (ACACTTGT). Thus, *LOC_Os05g32820* is a possible candidate gene for *qHGR5.2*.

## 3. Discussion

Direct rice seeding is less costly and labor-intensive in comparison to the traditional method of transplanting seedlings, thanks to its straightforward nature [39]. Given that the germination of rice seeds sown via this approach is vulnerable in rain-fed, flood-prone, and irrigated ecosystems, enhancing traits related to aerobic germination (AG) is essential for the shift from seedling transplantation to direct seeding [40]. Therefore, direct-seeded rice should possess relatively strong hypoxic germination ability and submergence tolerance of seedlings. Under the condition of being submerged in 16 cm of water, the germination rate and seedling establishment rate of Dongxiang wild rice can both reach over 90% (Figure 1A–F). Moreover, when the seedlings are submerged in 75 cm of water for as long as 15 days, the survival rate can reach 65% (Figure 1G). This indicates that Dongxiang wild rice has extremely strong flood tolerance. However, currently, most of the research on rice flood tolerance focuses on cultivated rice resources, making it difficult to break through the current bottleneck of flood tolerance in cultivated rice [6,7,8]. This study, based on the genetic mechanism of submergence tolerance in DXWR, is of great significance for the genetic improvement in submergence tolerance in cultivated rice.

There are various phenotypic evaluations of rice tolerance to flooding, including anaerobic response index, germ sheath length, anoxic response index, anaerobic germination, seedling survival rate, coleoptile length, etc. [18,19,20,22,23]. However, for direct rice seeding, the final hypoxic seedling rate and seedling survival rate under flooding should be the key to direct seeding. Rice direct seeding can be carried out using seeds in either dry or wet conditions. In order to systematically identify QTLs that can be used for the improvement in rice direct seeding, this study evaluated the anaerobic seedling establishment rates of both dry and wet rice seeds, as well as the subsequent survival rates of seedlings under submergence. A total of 20 QTLs were detected for the four flood tolerance traits, and 85% of them were minor QTLs (with a phenotypic variance explained (PVE) less than 8%) (Table 1). These QTLs did not include the previously reported major genes related to submergence tolerance. This can offer new ideas for the genetic improvement in rice submergence tolerance, and at the same, it also indicates that DXWR has a unique and complex mechanism for submergence tolerance.

**Figure 6:**
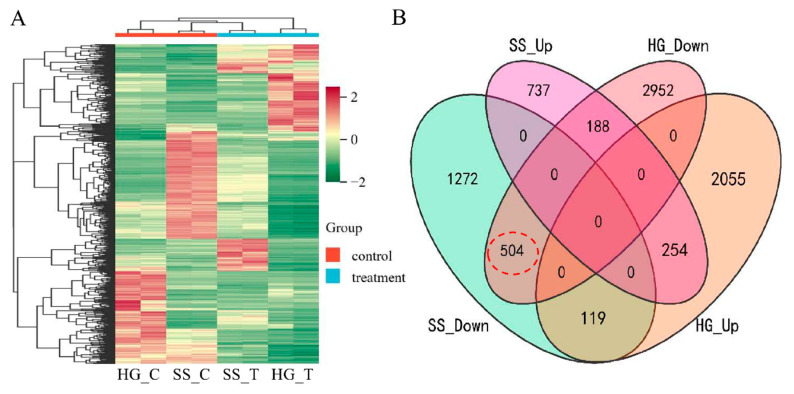
Joint analysis of differential expression genes (DEGs) between hypoxic germination and seedling flooding in DXWR. (**A**) The DEGs patterns detected by transcriptome sequencing before and after flooding treatment in DXWR at different stages. The red color represents higher expression and the green color represents lower expression. HG_C, HG_T, SS_C and SS_T represent the control sample of hypoxic germination, the treatment sample of hypoxic germination, the control sample of seedling flooding treatment and the treatment sample of seedling flooding, respectively. (**B**) are Venn diagram of DEGs between hypoxic germination and seedling flooding in DXWR. SS_Up and SS_Down represent the genes up-regulated and down-regulated before and after flooding of seedlings, respectively. HG_Down and HG_Up represent the genes up-regulated and down-regulated before and after hypoxic germination, respectively.

Transcriptome sequencing technology was utilized to analyze the genes that might be involved in the regulation of submergence tolerance in DXWR at the transcriptional level. A total of 6072 DEGs were detected during the hypoxic germination period, while only 3074 DEGs were found in seedlings under submergence (Figure 3A). This indicates that hypoxic germination has a more complex regulatory mechanism than the submergence of seedlings. In light of the remarkable correlation between hypoxic germination and seedling submergence (Figure S1), along with the presence of co-localized QTLs in both (Figure 2), it is consequently postulated that they share a common genetic underpinning. A joint analysis was performed on the DEGs detected in hypoxic germination and seedling submergence. It was found that, compared with the differences between different tissues, samples under submergence treatment had a more similar expression pattern (Figure 6A). The ensemble analysis of DEGs in hypoxic germination and seedling flooding showed that 254 genes were up-regulated and 504 genes were down-regulated simultaneously in hypoxic germination and seedling flooding (Figure 6B), indicating that down-regulation of gene expression is the main inherited mechanism of flooding regulation. UDP-glycosyltransferase activity was highly significantly enriched among the differentially expressed genes in both hypoxic germination and seedling submergence (Figure 3B and Figure 4B), and *OsUGT75A* has been reported to enhance rice flood tolerance by regulating the balance of JA and ABA [15], which corroborates that UDP-glycosyltransferase activity is an important pathway for regulating rice flood tolerance.

The combination of QTL-mapping and transcriptome analysis, in contrast to the methods of identifying candidate genes that rely solely on traditional QTL-mapping or high-throughput expression profiling, consumes less time, cuts down labor costs, and boosts the accuracy of selection for target regions or candidate genes [41,42]. In this study, a QTL that regulates HGR and SSSR was co-localized within a 347-kb interval. Only two genes within this interval had differential expression in the same trend during hypoxic germination and seedling submergence. One of them had no coding information, and the other gene, *LOC_Os05g32820*, which encodes PNAase A, was significantly down-regulated simultaneously during hypoxic germination and seedling submergence (Figure 5A,C,D). An insertion containing the *bZIP*-binding element was present in the promoter of *LOC_Os05g32820^GZX49^*. This has been reported numerous times on the regulation of rice stress resistance by the *bZIP* family [43,44,45]. This assumption offers a novel perspective for the subsequent research on the genetic mechanism underlying submergence tolerance of DXWR.

## 4. Materials and Methods

### 4.1. Plant Materials

A backcross recombinant inbred line (BRIL) population consisting of 132 lines was used for phenotype investigation in submergence tolerance tests, and its construction process and genotype have been previously reported [37]. The BRIL population was grown at the experimental field of Jiangxi Academy of Agricultural Sciences in 2019 at Nanchang (28.57 N, 115.9 E), China. Field management was consistent with local field production.

### 4.2. Evaluation of Submergence Tolerance

Select fresh and plump DXWR seeds that have broken dormancy, place 30 seeds in a glass bottle with a diameter of 3.5 cm and a depth of 16 cm, fill it with distilled water, and cultivate under a 12 h light/12 h dark cycle at 28 °C/26 °C. After 96 h, the germination rate of the seeds was calculated. Those with a length of 0.5 cm were considered germinated, while others were considered ungerminated. The hypoxic germination rate was calculated based on this standard. Under the same growth conditions, count the number of seedlings formed after 15 days. Those containing green embryo sheaths were considered normal seedlings, while others were considered failed seedlings. The seedlings submergence survival rate was calculated based on this standard.

The harvested BRIL seeds were incubated at 40 °C for approximately 36 h to break dormancy and soaked in deionized water at 30 °C for approximately 60 h for germination. Pick 30 germinated seeds in a glass bottle with a diameter of 3.5 cm and a depth of 16 cm, fill it with distilled water, and cultivate under a 12 h light/12 h dark cycle at 28 °C/26 °C. The number of seedlings formed after 15 days was counted, and the budlet submergence survival rate was calculated.

A total of 30 germinated seeds from each line were selected and sown in soil in pots and cultivated under a 12 h light/12 h dark cycle at 28 °C/26 °C with 80% humidity. When most of the seedlings grew to one leaf and one core, weak seedlings were removed, and the remaining seedlings were moved to a white plastic bucket with a diameter of 75 cm and a depth of 1 m, filled with distilled water for 15 days submergence treatment. Then, they were allowed to resume growth under normal conditions for 7 days, and the surviving plants (leaf contains about 40% green part) and dead plants were investigated, and the survival rate was calculated. The seedlings submergence survival rate was calculated based on this standard.

All lines were subjected to three independent replicates.

### 4.3. QTL Analysis

The QTL analysis of the phenotypic data with bin-maps in the BILs was performed using the linear ridge regression method to reduce the multicollinearity among markers as described previously [46]. A significance level of *p* < 0.05 was set as the threshold in the BILs to declare the presence of a putative QTL in a given bin. If several adjacent bins showed *p* values lower than the threshold, the QTL was tentatively located in the bin (peak bin) with the lowest *p* value [46]. The phenotypic variance explained by each QTL was decomposed using the “relaimpo” package of R (R version 4.3.3) (“lmg” function). QTL nomenclature followed the principles suggested by a previous report [47].

Gene annotations for a given peak bin were obtained from the Rice Genome Annotation Project Database (http://rice.plantbiology.msu.edu/ (accessed on 28 March 2024)).

### 4.4. RNA-Seq Analysis

Select fresh and plump DXWR seeds that break dormancy, place them in a glass bottle with a diameter of 3.5 cm and a depth of 16 cm, fill it with distilled water, and cultivate under a 12 h light/12 h dark cycle at 28 °C/26 °C. Take 6 h of germination as a blank control sample and 24 h of germination as an anaerobic treatment sample. Use these samples for analyzing the transcriptome of hypoxic germination in DXWR.

A total of 30 germinated seeds from each line were selected and sown in soil in pots and cultivated under a 12 h light/12 h dark cycle at 28 °C/26 °C with 80% humidity. When most of the seedlings grew to one leaf and one core, weak seedlings were removed, and the remaining seedlings were moved to a white plastic bucket with a diameter of 75 cm and a depth of 1 m, filled with distilled water, for submergence treatment. Samples taken before submergence were used as blank controls, and samples taken after 48 h of flooding were used as samples for seedling flooding treatment. These samples were used for analyzing the transcriptome of seedlings submergence in DXWR.

Eight RNA samples, each sample including submergence treatment and controls in two replicates, were used for RNA sequencing. A total amount of 1 µg RNA per sample was used as input material for the RNA sample preparations. Sequencing libraries were generated using NEBNext^®^ UltraTM RNA Library Prep Kit for Illumina^®^ (NEB, Ipswich, MA, USA) following manufacturer’s recommendations and index codes were added to attribute sequences to each sample. Briefly, mRNA was purified from total RNA using poly-T oligo attached magnetic beads. Fragmentation was carried out using divalent cations under elevated temperature in NEBNext First Strand Synthesis Reaction Buffer (5×). First, strand cDNA was synthesized using random hexamer primer and M-MuLV Reverse Transcriptase (RNase H-). Second strand cDNA synthesis was subsequently performed using DNA Polymerase I and RNase H. The remaining overhangs were converted into blunt ends via exonuclease/polymerase activities. After adenylation of 3′ ends of DNA fragments, NEBNext Adaptor with hairpin loop structure was ligated to prepare for hybridization. In order to select cDNA fragments of preferentially 250~300 bp in length, the library fragments were purified with AMPure XP system (Beckman Coulter, Beverly, MA, USA). Then, 3 µL USER Enzyme (NEB, USA) was used with size-selected, adaptor-ligated cDNA at 37 °C for 15 min followed by 5 min at 95 °C before PCR. Then, PCR was performed with Phusion High-Fidelity DNA polymerase, universal PCR primers and Index (X) Primer. At last, PCR products were purified (AMPure XP system) and library quality was assessed on the Agilent Bioanalyzer 2100 system.

Then, fastp v 0.19.3 was used to filter the original data, mainly to remove reads with adapters. When the N content in any sequencing reads exceeds 10% of the base number of the reads, the paired reads are removed. When the number of low-quality (Q ≤ 20) bases contained in reads exceeds 50% of the bases of the reads, these paired reads are removed. All subsequent analyses were based on clean reads.

The reads were aligned to the reference transcript sequence (http://rice.plantbiology.msu.edu/ (accessed on 8 June 2024)), and the number of reads covered from the start to the end of each gene was counted. Used feature Counts v1.6.2 to calculate the gene alignment, and then calculate the FPKM of each gene based on the gene length. FPKM is currently the most commonly used method to estimate gene expression levels. DESeq2 v1.22.1 was used to analyze the differential expression between the two groups, and the *p*-value was corrected using the Benjamini and Hochberg method. The corrected *p* value and log2foldchange were used as the threshold for significant difference expression. The enrichment analysis was performed based on the hypergeometric test. For KEGG, the hypergeometric distribution test was performed with the unit of pathway; for GO, it was performed based on the GO term. The above RNA-seq-related experiments were completed by Wuhan Metware Bioinformatics Co., Ltd. (Wuhan, China). The original RNA-seq data set has been deposited in NCBI (PRJNA1201802).

### 4.5. Quantitative Real-Time PCR

Total RNAs were isolated with the TRIzol kit (Invitrogen, Carlsbad, CA, USA) according to the manufacturer’s instructions. The RNA was treated with DNase I (Invitrogen), and approximately 3 μg of total RNA was used to synthesize first-strand cDNA using oligo (dT)_18_ as a primer (Promega, Shanghai, China). The trans-intron ACTIN primer (ACTIN-M) was used to detect whether the reverse-transcribed cDNA still has genomic DNA, and the cDNA without genomic DNA was used for subsequent quantitative real-time PCR. Quantitative real-time (qRT) PCR was performed using gene-specific primers and the FastStart Universal SYBR Green Master (Roche) on a real-time PCR ViiA7 system (Applied Biosystems). Genes *ubiquitin* (*LOC_Os03g13170*) and *actin1* (*LOC_Os03g50885*) not differentially expressed in RNAseq were used as the internal control. The relative quantification method (2^−△△CT^) was used to evaluate gene expression level [48]. As similar expression results were observed regardless of which control genes were used, the *ubiquitin* of expressions was applied for the relative expression analyses for every assayed sample. At least three biological replicates, each containing four technical aspects, were performed for each experiment.

The primers were designed according to the Nipponbare reference genome by Primer3 (RiceVarMap2 (ncpgr.cn) http://ricevarmap.ncpgr.cn/primer_by_region/ (accessed on 18 April 2024)). The sequences were analyzed using Sequencer 5.0 (Gene Codes Corporation). All primers were synthesized at Sangon Biotech (Shanghai, China) and are listed in Table S1.

## 5. Conclusions

In summary, DXWR exhibits robust submergence tolerance. The BRIL population of DXWR/GZX49, accompanied by a high-density bin map, was employed to detect several QTLs and potential candidate genes associated with submergence tolerance. Transcriptome sequencing was utilized to identify submergence-responsive genes in DXWR. Additionally, through the integration of transcriptome data and genetic linkage analysis, sequence comparison and bioinformatics, the candidate gene of HGR and SSSR co-localized locus *qHGR5.2*, namely *LOC_Os05g32820*, was determined. These discoveries offer valuable perspectives into the genetic foundation of submergence tolerance, which is beneficial for enhancing submergence tolerance in rice breeding initiatives.

## Figures and Tables

**Table 1:** QTLs detected for submergence tolerance in BRIL population.

Traits ^1^	*QTL*	Bin	Chr	Bin_Start (bp)	Bin_End (bp)	Bin Length (bp)	Effect	*p* Value	PVE
HGR	*qHGR3*	bin0515	3	21,007,100	21,095,363	88,263	0.029	6.05 × 10^−3^	6.5%
HGR	*qHGR5.1*	bin0744	5	2,255,061	3,013,554	758,493	−0.029	7.22 × 10^−3^	4.9%
HGR	*qHGR5.2*	bin0806	5	19,199,634	19,265,882	66,248	0.039	3.26 × 10^−4^	8.4%
HGR	*qHGR10*	bin1332	10	2,724,466	3,462,816	738,350	−0.036	3.85 × 10^−4^	6.8%
HGR	*qHGR12*	bin1579	12	3,383,694	3,538,969	155,275	0.029	4.77 × 10^−3^	5.0%
BSSR	*qBSSR1*	bin0032	1	6,622,905	6,688,501	65,596	0.079	4.27 × 10^−3^	5.5%
BSSR	*qBSSR3.1*	bin0488	3	16,678,957	16,783,042	104,085	0.091	2.23 × 10^−3^	5.2%
BSSR	*qBST3.2*	bin0530	3	22,817,119	22,945,933	128,814	0.082	4.01 × 10^−3^	4.3%
BSSR	*qBSSR5*	bin0784	5	15,986,828	16,044,287	57,459	0.079	3.69 × 10^−3^	4.9%
BSSR	*qBSSR9*	bin1252	9	9,245,327	9,308,475	63,148	0.072	6.88 × 10^−3^	4.2%
HSR	*qHSR1.1*	bin0023	1	4,731,000	4,833,001	102,001	0.089	2.45 × 10^−3^	5.3%
HSR	*qHSR1.2*	bin0079	1	18,829,703	20,016,518	1,186,815	0.09	1.78 × 10^−3^	5.3%
HSR	*qHSR2*	bin0267	2	10,660,601	10,871,055	210,454	−0.076	4.72 × 10^−3^	3.3%
HSR	*qHSR3*	bin0552	3	26,754,226	26,796,730	42,504	−0.048	8.49 × 10^−3^	2.0%
HSR	*qHSR8*	bin1099	8	537,935	577,294	39,359	0.072	5.01 × 10^−3^	4.5%
SSSR	*qSSSR2*	bin0315	2	21,421,270	21,586,259	164,989	0.03	7.07 × 10^−3^	4.8%
SSSR	*qSSSR3*	bin0481	3	15,048,212	15,219,822	171,610	0.035	7.89 × 10^−4^	8.4%
SSSR	*qSSSR4*	bin0657	4	15,456,730	15,899,273	442,543	0.044	4.68 × 10^−3^	7.3%
SSSR	*qSSSR5*	bin0807	5	19,265,882	19,545,899	280,017	0.028	9.18 × 10^−3^	3.6%
SSSR	*qSSSR6*	bin0997	6	27,583,792	27,878,278	294,486	−0.056	2.14 × 10^−4^	8.5%

^1^ HGR, BSSR, HSR and SSSR represents hypoxic germination rate, budlet submergence survival rate, hypoxic seedling rate and seedling submergence survival rate, respectively.

## Data Availability

Data is contained within the article and Supplementary Materials.

## Supplementary Materials

The supporting information can be downloaded at: https://www.mdpi.com/article/10.3390/ijms26051829/s1.
