# Supplementary material for: Identification of Submergence Tolerance Loci in Dongxiang Wild Rice (DXWR) by Genetic Linkage and Transcriptome Analyses
Source: Int J Mol Sci. 2025 Feb 20;26(5):1829. doi: 10.3390/ijms26051829 (PMC11898957; doi:10.3390/ijms26051829)
Supplement: Supplementary file 1 [file ijms-26-01829-s001.zip › ijms-3404640-supplementary.pdf]

Supplemental data

1. Supplemental Figures

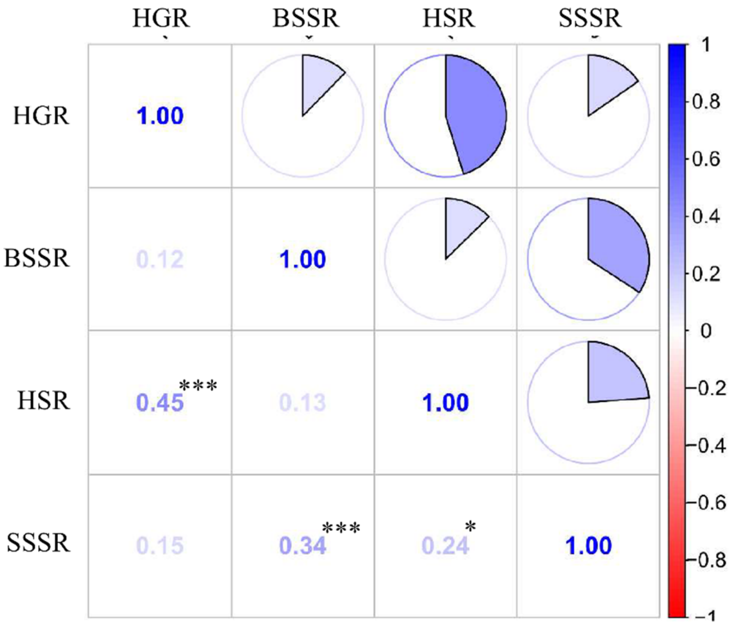

Figure S1. Correlation analysis of four submergence tolerance related phenotypes in the BRIL population. HGR, BSSR, HSR, and SSSR respectively represent hypoxic germination rate, budlet submergence survival rate, hypoxic seedling rate and seedling submergence survival rate. The number in the bottom left corner represents the correlation coefficient, and the asterisk on the number indicates the significance of the correlation. * and *** respectively represent the significance levels of 0.05 and 0.001.

2. Supplemental Tables

Table S1. Primers used in the study.

Primer	Forward primer sequence (5'-3')	Purpose
Ubq-qRT-F	AACCAGCTGAGGCCCAAGA	qRT-PCR, Reference gene
Ubq-qRT-R	ACGATTGATTTAACCAGTCCATGA	
ACTIN1-qRT-F	ACATCGCCCTGGACTATGACCA	qRT-PCR, Reference gene
ACTIN1-qRT-R	GTCGTACTCAGCCTTGGCAAT	
LOC_Os05g32820-RTF	GAATCCAGGTGAAATTCGTCAG	qRT-PCR, target gene
LOC_Os05g32820-RTR	AAAAGCAATAGTCTTGGTCACG	
actin-M-F	CTCAACCCCAAGGCTAACAG	cDNA detection
actin-M-R	ACCTCAGGGCATCGGAAC	
LOC_Os05g32820POF	CGCCGGAGGACTTCGCCGTGG	PCR amplification and sequencing
LOC_Os05g32820POR	TGTCGCCGAAGGAGTAGGAGAGGA	
LOC_Os05g32820POSQ1	CCCTTTTGAGTCAAAGGGAATT	
LOC_Os05g32820POSQ2	ATCAGGTGTGAATCACGGCTCCA	
LOC_Os05g32820PTF	ATGGCGCCGCCGCTCTATCTCCT	
LOC_Os05g32820PFR	ATGCGGTCGGAAACGAATCCTGC	
LOC_Os05g32820PTSQ1	AAGGAACCTATGCTGCCGGAGT	
